# Reviewer summary for journal of arrhythmia

**DOI:** 10.1002/joa3.70090

**Published:** 2025-06-05

The Editorial Board members of the Journal of Arrhythmia are grateful to the following reviewers who provided their expertise and knowledge to the journal.

Fukaya, Hidehira

hidehira@med.kitasato-u.ac.jp

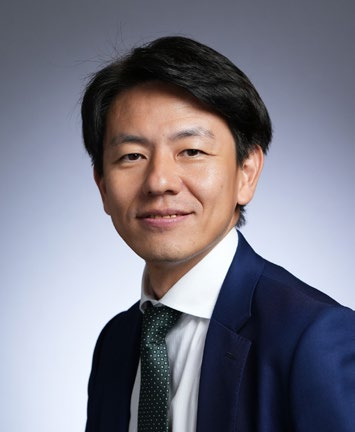

Oginosawa, Yasushi

y-ogi@med.uoeh-u.ac.jp

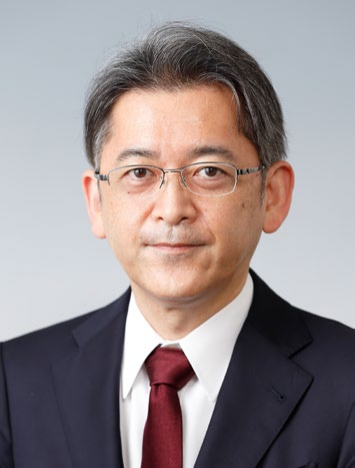

Nishii, Nobuhiro

nnishii@md.okayama-u.ac.jp

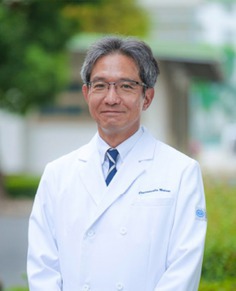

Inden, Yasuya

Mukai, Yasushi

Ishibashi, Kohei

Joung, Boyoung

Yoshida, Kentaro

Kaneko, Yoshiaki

Kimura, Masaomi

Morishima, Itsuro

Nakahara, Shiro

Sato, Toshiaki

Wada, Mitsuru

Aizawa, Yoshiyasu

Ching, Chi Keong

Ikeda, Yoshifumi

Arimoto, Takanori

Kataoka, Naoya

Maruyama, Mitsunori

Nakai, Toshiko

Noda, Takashi

Tsutsui, Kenta

Chen, Wei‐Ta

Hasegawa, Kanae

Hayashi, Tatsuya

Irie, Tadanobu

Kuroki, Kenji

Sasaki, Shingo

Shimojo, Masafumi

Shinohara, Tetsuji

Tobiume, Takeshi

Yanagisawa, Satoshi

Ejima, Koichiro

Fukuzawa, Koji

Higa, Satoshi

Kodani, Eitaro

Mizutani, Yoshiaki

Mori, Hitoshi

Nabeshima, Taisuke

Nagashima, Koichi

Naruse, Yoshihisa

Sekihara, Takayuki

Shiga, Tsuyoshi

Sobue, Yoshihiro

Yamasaki, Hiro

Aizawa, Yoshifusa

Canpolat, Uğur

Chang, Shih‐Lin

Hachiya, Hitoshi

Inoue, Koichi

Iwasaki, Yuki

Kamakura, Tsukasa

Kanzaki, Yasunori

Komatsu, Yuki

Kondo, Yusuke

Matsunaga‐Lee, Yasuharu

Miyazaki, Aya

Nakatani, Yosuke

Ogawa, Masahiro

Ohe, Masatsugu

Takenaka, Sou

Yodogawa, Kenji

Yokoshiki, Hisashi

Fukunaga, Masato

Hashimoto, Kenichi

Hayashi, Kentaro

Hori, Yuichi

Kabutoya, Tomoyuki

Kajiyama, Takatsugu

Kanaoka, Koshiro

Kaneko, Shinji

Kaneshiro, Takashi

Kumagai, Koji

Masuda, Masaharu

Matsumoto, Kazuhisa

Miyamoto, Koji

Nagase, Takahiko

Nakasuka, Kosuke

Nam, Gi‐Byoung

Sakamoto, Yuichiro

Takahashi, Yoshihide

Tanno, Kaoru

Tonegawa‐Kuji, Reina

Ueda, Akiko

Watanabe, Atsuyuki

Yagi, Tetsuo

Yamashita, Seigo

Calvert, Peter

Fukamizu, Seiji

Furusho, Hiroshi

Hayashi, Meiso

Higuchi, Satoshi

Inaba, Osamu

Kawaji, Tetsuma

Kawakami, Hiroshi

Kawamura, Mitsuharu

Kobori, Atsushi

Kusa, Shigeki

Lau, Dennis

Mari, Amino

Matsumoto, Katsumi

Murase, Yosuke

Nagase, Satoshi

Nakajima, Ikutaro

Nakamura, Tomofumi

Oka, Takafumi

Osanai, Hiroyuki

Sasano, Tetsuo

Shimamoto, Keiko

Suzuki, Hirohiko

Suzuki, Shinya

Suzuki, Tsugutoshi

Togashi, Ikuko

Tokuda, Michifumi

Wakamiya, Akinori

Yasuoka, Ryobun

Yokoyama, Yasuhiro

Yoshiga, Yasuhiro

Amaya, Naoki

An, Yoshimori

Asano, Taku

Ashihara, Takashi

Chinushi, Masaomi

Fujiu, Katsuhito

Goto, Koji

Hara, Satoshi

Harada, Masahide

Heeger, Christian

Hong, Kui

Imai, Yasushi

Inaba, Hideo

Ishizawa, Makoto

John, Roy

Kato, Hiroyuki

Kato, Yoshiaki

Kim, Daehoon

Kiuchi, Kunihiko

Kondo, Hidekazu

Liao, Ying‐Chieh

Lo, Li‐Wei

Makimoto, Hisaki

Mazzone, Patrizio

Minamiguchi, Hitoshi

Mine, Takanao

Miyanaga, Satoru

Miyauchi, Yasushi

Miyazaki, Shinsuke

Morita, Junji

Morita, Norishige

Murakami, Masato

Nagata, Yasutoshi

Nodera, Minoru

Nozoe, Masatsugu

Ogano, Michio

Sano, Makoto

Sekiguchi, Yukio

Sternick, Eduardo Back

Sunaga, Akihiro

Tabuchi, Haruna

Tanaka, Yasuaki

Tateishi, Ryo

Temma, Taro

Terata, Ken

Tsurugi, Takuo

Yamabe, Hiroshige

Yamaguchi, Takanori

Yasuhiro, Ogura

Yoshida, Akira

Akao, Masaharu

Aoki, Hisaaki

Chang, Ting‐Yung

Chung, Fa‐Po

Fujimoto, Yuhi

Fukuda, Koji

Fukue, Noriko

Goto, Toshihiko

Gupta, Dhiraj

Hayashi, Kenshi

Hiroshima, Ken‐Ichi

Hojo, Rintaro

Horie, Minoru

Igarashi, Miyako

Imai, Katsuhiko

Imamura, Teruhiko

Inamura, Yukihiro

Inoue, Shujiro

Inoue, Yuko

Ito‐Hagiwara, Kanako

Kato, Takeshi

Kim, Jun

Kinoshita, Toshio

Kohno, Ritsuko

Kojima, Toshiya

Kuo, Jen‐Yuan

Kurita, Takashi

Kutarski, Andrzej

Latcu, Gabriel

Maruyama, Masahiro

Nakajima, Kenzaburo

Nakamura, Kohki

Nakamura, Toshihiro

Nakamura, Yuya

Nakano, Yukiko

Nishida, Taku

Nishiuchi, Suguru

Nishiyama, Nobuhiro

Niwano, Shinichi

Okada, Ayako

Okubo, Kenji

Okubo, Yousaku

Ono, Morio

Otsuki, Sou

Ozawa, Tomoya

Romiti, Giulio Francesco

Saito, Jumpei

Sasaki, Takeshi

Seo, Yoshihiro

Shinohara, Masaya

Shizuta, Satoshi

Soeki, Takeshi

Suzuki, Atsushi

Suzuki, Makoto

Takagi, Masahiko

Takase, Bonpei

Takemoto, Masao

Takeuchi, Daiji

Tao, Susumu

Tokano, Takashi

Tsuboi, Ippei

Uetake, Shunsuke

Wada, Tadashi

Yamane, Teiichi

Yano, Masamichi

Yoshimura, Sohei

Yoshioka, Koichiro

Yu, Chih‐Chieh

Abe, Ichitaro

Ahn, Hyo‐Jeong

Anand, Abhinav

Ando, Monami

Bilchick, Kenneth C. C

Boriani, Giuseppe

Chao, Tze‐Fan

Chen, Minglong

Cheng, Wen‐Han

Chiladakis, John

Choi, Jong‐Il

Chrishan, Nalliah

Doshi, Rushabh

Enomoto, Yoshinari

Fan, Xiaohan

Fujino, Tadashi

Fukui, Akio

Gatzoulis, Konstantinos

Gawałko, Monika

Harada, Tomoo

Hasebe, Hideyuki

Hayashi, Hiroshi

Hayashi, Katsuhide

Hirata, Shu

Horigome, Hitoshi

Hu, Yu‐Feng

Ichiro, Sakuma

Jastrzebski, Marek

Kamihara, Takahiro

Kanazawa, Hisanori

Kasai, Takatoshi

Kawamura, Iwanari

Keida, Takehiko

Kimura, Takehiro

Korantzopoulos, Panagiotis

Kowase, Shinya

Kuan‐Hung, Yeh

Kumagai, Koichiro

Kurasawa, Yasuyuki

Kuwahara, Taishi

Kwon, Younghoon

Lin, Lian‐Yu

Lin, Wei‐Shiang

Lin, Wen‐Yu

Lokhandwala, Yash

Mahajan, Rajiv

Malik, Varun

Marine, Joseph E. E

Maruyama, Toru

Matsuda, Yasuhiro

Matsuo, Seiichiro

Matsuyama, Takaaki

Mitsuhashi, Takeshi

Miyajima, Keisuke

Mizukami, Akira

Morita, Hiroshi

Morris, Gwilym

Morton, Joseph

Nagashima, Michio

Nakagawa, Koji

Nakamura, Kazufumi

Nakamura, Keijiro

Nakano, Makoto

Nakano, Masahiro

Namboodiri, Narayanan

Nantsupawat, Teerapat

Narita, Masataka

Narita, Yuji

Noda, Kazuki

Odagiri, Fuminori

Oh, Il‐Young

Oka, Satoshi

Onishi, Yoshimi

Otsubo, Toyokazu

Park, David S

Pathak, Rajeev

Phrommintikul, Arintaya

Prasertwitayakij, Narawudt

Sahin, Irfan

Sairaku, Akinori

Santangeli, Pasquale

Sato, Hiroyuki

Shehata, Islam

Shiozawa, Tomoyuki

Shirai, Shinichi

Shirai, Yasuhiro

Shiraishi, Hirokazu

Siontis, Konstantinos

Stiles, Martin

Sugiyama, Atsushi

Sumitomo, Naokata

Suzuki, Yasushi

Takahashi, Naohiko

Takasugi, Nobuhiro

Takatsuki, Seiji

Teo, Wee‐Siong

Tomii, Naoki

Tsai, Chia‐Ti

Tsai, Chin‐Feng

Tsuji, Yukiomi

Ueda, Nobuhiko

Uhm, Jae‐Sun

Vijay, Jyothi

Wakamatsu, Yuji

Watanabe, Masaya

Watanabe, Ryuta

Wijayanto, Matthew Aldo

Wilsmore, Bradley

Wong, Christopher

Yagishita, Atsuhiko

Yamada, Shinya

Yamagata, Kenichiro

Yamaguchi, Osamu

Yamaji, Hirosuke

Yamamoto, Teppei

Yamashiro, Kohei

Yazaki, Kyoichiro

Yinadsawaphan, Thanaboon

Yokoyama, Teruki

